# Supplementary figures and images for: Rapid preparation of terbium-doped titanium dioxide nanoparticles and their enhanced photocatalytic performance
Source: R Soc Open Sci. 2019 Oct 9;6(10):191077. doi: 10.1098/rsos.191077 (PMC6837207; doi:10.1098/rsos.191077)

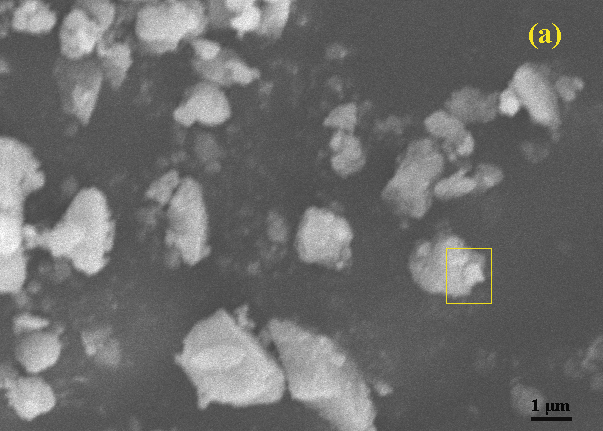

Supplement: Fig. 3 [file rsos191077supp3.zip › Fig.3(a).tif]

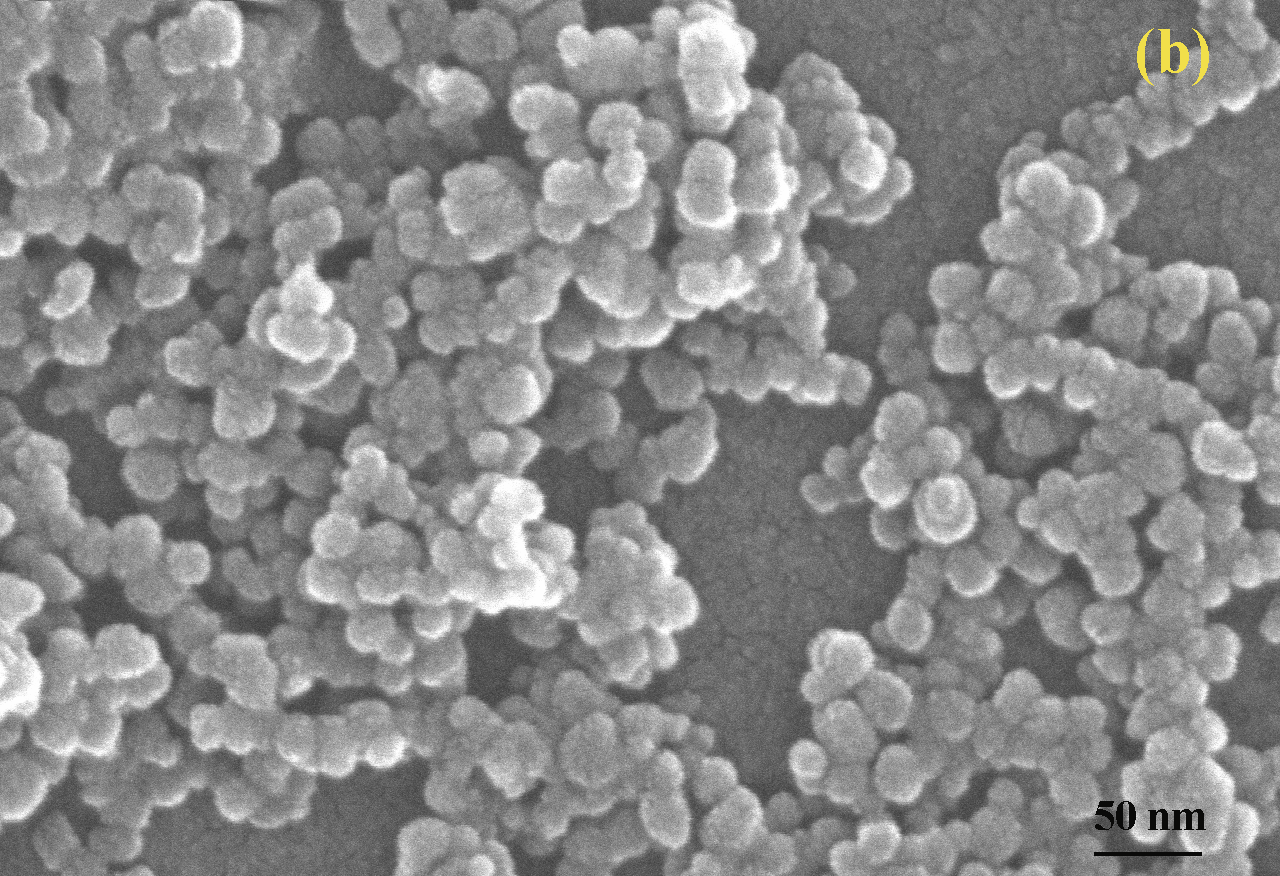

Supplement: Fig. 3 [file rsos191077supp3.zip › Fig.3(b).tif]

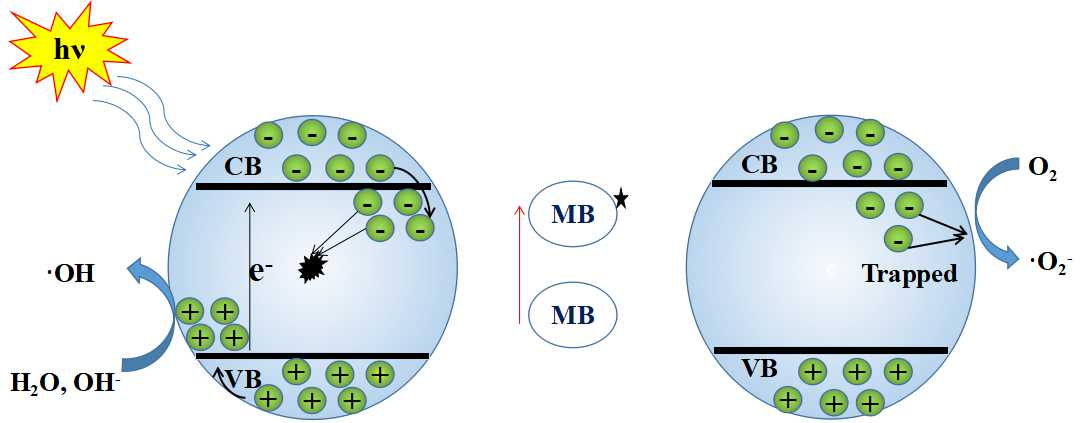

Supplement: Fig. 13 [file rsos191077supp13.jpg]
