# Supplementary material for: Rapid preparation of terbium-doped titanium dioxide nanoparticles and their enhanced photocatalytic performance
Source: R Soc Open Sci. 2019 Oct 9;6(10):191077. doi: 10.1098/rsos.191077 (PMC6837207; doi:10.1098/rsos.191077)
Supplement: Table 1 [file rsos191077supp14.docx]

**Table 1** Parameters of the samples

| Samples | Exact doping amount of Tb (%) | Crystallinity (%) | Crystal size (nm) |
| --- | --- | --- | --- |
|  |  |  |  |
| Pure TiO_2_ | 0 | 99.69 | 12.7 |
| 0.5%Tb-TiO_2_ | 0.4996 | 98.76 | 11.8 |
| 1.0%Tb-TiO_2_ | 0.9899 | 99.18 | 10.8 |
| 1.5%Tb-TiO_2_ | 1.4972 | 95.31 | 10.4 |
| 2.0%Tb-TiO_2_ | 2.0046 | 97.51 | 10.1 |
