# Supplementary material for: Rapid preparation of terbium-doped titanium dioxide nanoparticles and their enhanced photocatalytic performance
Source: R Soc Open Sci. 2019 Oct 9;6(10):191077. doi: 10.1098/rsos.191077 (PMC6837207; doi:10.1098/rsos.191077)
Supplement: Table 2 [file rsos191077supp15.docx]

**Table 2** Results of zebrafishes acute toxicity tested by Tb-TiO_2_

| Catalyst concentration (mg·L^-1^) | 2h | 6h | 24h | 48h | 72h | 96h |
| --- | --- | --- | --- | --- | --- | --- |
| 0 | A | A | A | A | A | A |
| 12.5 | A | A | A | A | A | O, B, I |
| 25 | A | A | A | O, B, I | O, B, I | P |
| 50 | A | O, B, I | O, B, I | P | P | P |
| LC_50_ (mg·L^-1^) | ---- | ---- | 41.6 | 32.8 | 27.3 | 23.2 |
| 95% confidence interval (mg·L^-1^) | ---- | ---- | 39.6-43.6 | 31.1-34.5 | 25.5-28.1 | 21.9-24.5 |

A. Normal; B. Swimming retardation; I. Breathe weakness; O. Afloat; P. All death．
